# Tacrolimus’s Time Below Therapeutic Range Is Associated With Acute Pancreatic Graft Rejection and the Development of *De Novo* Donor-specific Antibodies

**DOI:** 10.3389/ti.2024.12591

**Published:** 2024-04-17

**Authors:** Diana Rodríguez-Espinosa, José Jesús Broseta, Enrique Montagud-Marrahí, Carolt Arana, Joana Ferrer, Miriam Cuatrecasas, Ángeles Garcia-Criado, Antonio J. Amor, Fritz Diekmann, Pedro Ventura-Aguiar

**Affiliations:** ^1^ Department of Nephrology and Renal Transplantation, Hospital Clínic de Barcelona, Barcelona, Spain; ^2^ Laboratori Experimental de Nefrologia I Trasplantament (LENIT), Institut d’Investigacions Biomèdiques August Pi i Sunyer (IDIBAPS), Barcelona, Spain; ^3^ Department of Hepatobiliopancreatic Surgery and Liver Transplant, Hospital Clínic, Barcelona, Spain; ^4^ Department of Pathology, Hospital Clínic Barcelona, Barcelona, Spain; ^5^ Department of Radiology, Hospital Clínic Barcelona, Barcelona, Spain; ^6^ Diabetes Unit, Department of Endocrinology and Nutrition, Hospital Clínic Barcelona, Barcelona, Spain

**Keywords:** pancreas transplantation, tacrolimus, FK, time in therapeutic range, TTR

## Abstract

Tacrolimus is pivotal in pancreas transplants but poses challenges in maintaining optimal levels due to recipient differences. This study aimed to explore the utility of time spent below the therapeutic range and intrapatient variability in predicting rejection and *de novo* donor-specific antibody (dnDSA) development in pancreas graft recipients. This retrospective unicentric study included adult pancreas transplant recipients between January 2006 and July 2020. Recorded variables included demographics, immunosuppression details, HLA matching, biopsy results, dnDSA development, and clinical parameters. Statistical analysis included ROC curves, sensitivity, specificity, and predictive values. A total of 131 patients were included. Those with biopsy-proven acute rejection (BPAR, 12.2%) had more time (39.9% ± 24% vs. 25.72% ± 21.57%, *p* = 0.016) and tests (41.95% ± 13.57% vs. 29.96% ± 17.33%, *p* = 0.009) below therapeutic range. Specific cutoffs of 31.5% for time and 34% for tests below the therapeutic range showed a high negative predictive value for BPAR (93.98% and 93.1%, respectively). Similarly, patients with more than 34% of tests below the therapeutic range were associated with dnDSA appearance (38.9% vs. 9.4%, *p* = 0.012; OR 6.135, 1.346–27.78). In pancreas transplantation, maintaining optimal tacrolimus levels is crucial. Suboptimal test percentages below the therapeutic range prove valuable in identifying acute graft rejection risk.

## Graphical Abstract

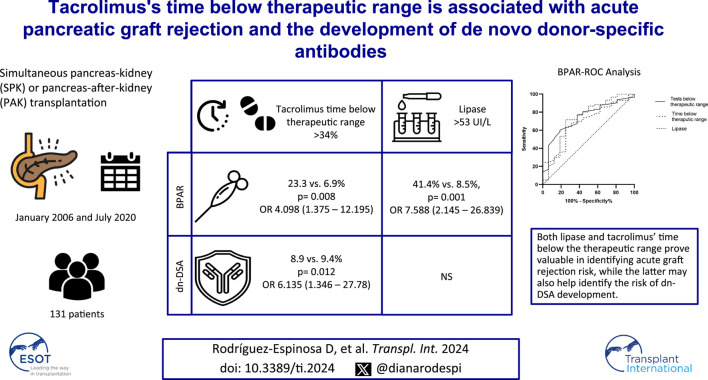

## Introduction

Tacrolimus has been the mainstay immunosuppressive agent in pancreas transplantation in the last two decades [1–3], given its effectiveness in preventing rejections and increasing graft survival [4]. It presents a narrow therapeutic window, requiring strict monitoring and constant dosing modification [5]. Differences in tacrolimus absorption [6, 7], metabolism [8, 9], and drug interactions [6, 10, 11] often lead to either sub- or supratherapeutic trough levels [12, 13]. Above-target trough levels are associated with adverse effects, whereas those below target are associated with an increased risk of rejection and development of *de novo* donor-specific antibody (dnDSA) [14, 15].

Given the pharmacokinetics and pharmacodynamics of tacrolimus [6, 8–11], several formulas have been developed to explore the correlation of tacrolimus trough levels with graft outcomes. Intrapatient variability (IPV) calculates the variability coefficient by dividing the standard deviation of tacrolimus samples by their mean [16, 17]. A high IPV has been associated with an increased risk of rejection, development of dnDSA, and graft failure in kidney transplantation [16–20] and with rejections in heart [21] and lung [22], though not in liver transplantation [23]. Time in therapeutic range (TTR), first developed by Rosendaal et al. to monitor anticoagulation time in patients on warfarin [24], has been recently used to explore the correlation of the time of tacrolimus within the therapeutic window and its correlation with graft outcomes. In lung, heart, and kidney transplantation, a lower TTR is associated with dnDSA development [25], acute rejection, and graft survival [26–29]. However, there are many concerns about the accuracy of this formula, as it assumes tacrolimus will change linearly from test to test [30]. The method used for managing warfarin assumes a linear increase or decrease between two consecutive INR (International Normalized Ratio) determinations [30, 31]. Therefore, we propose using the formula that calculates the ratio of samples within the therapeutic range to the total number of samples, also from Rosendaal et al. [24]. Additionally, if the primary study outcome is immunological, it may be more useful to only determine the time below the therapeutic range [25]. To date, the ability of these formulas to predict pancreas graft outcomes has yet to be explored.

In this study, we aimed to determine the utility of tacrolimus IPV and the time and test results below the therapeutic range in identifying the risks of rejection and dnDSA development for pancreatic grafts in recipients of pancreas transplants.

## Materials and Methods

### Patient Population

**FIGURE 1:**
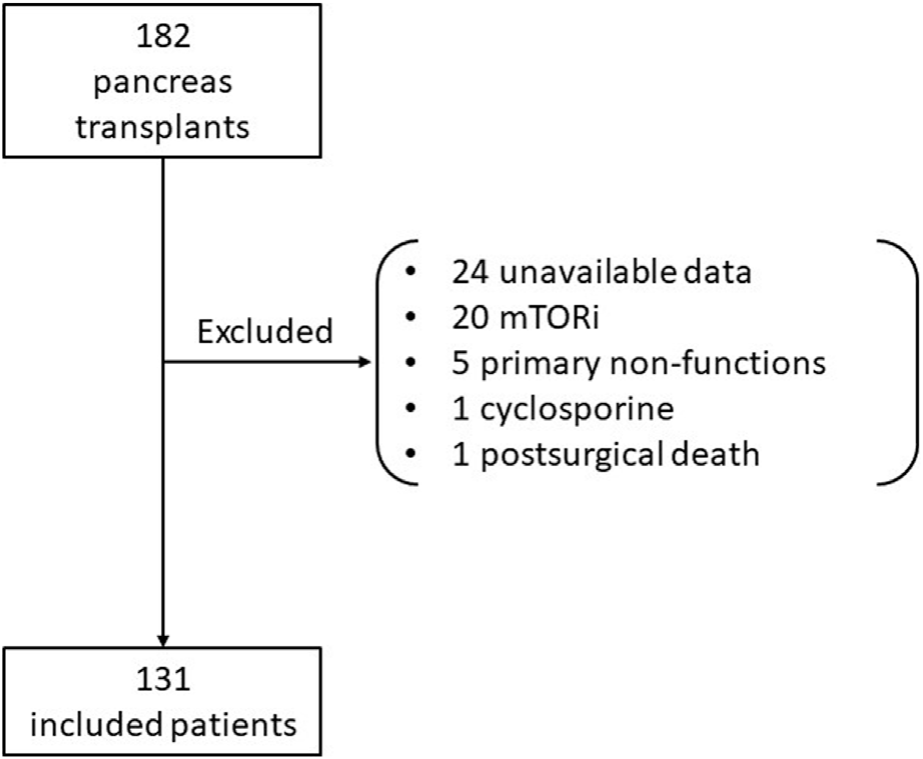
Flowchart of included patients.

We conducted a retrospective unicentric study including all adult pancreas transplant recipients between January 2006 and July 2020 from Hospital Clínic of Barcelona. Both simultaneous pancreas-kidney (SPK) and pancreas after kidney (PAK) were analyzed. We excluded patients in whom TTR was not possible to calculate; those who had a primary non-function pancreas graft, those lost to follow-up, and those who died due to transplantation surgery. One-hundred and eighty-two pancreas transplants were performed during the study period; fifty-one were excluded (Figure 1). Data was gathered from electronic clinical records. This study was conducted in accordance with the Declaration of Helsinki.

### Variables

Demographic data such as age, weight, body mass index, sex, and race at the time of transplantation were recorded for donors and recipients. Induction immunosuppression was performed with rabbit antithymocyte globulin (rATG) or basiliximab. Maintenance immunosuppression consisted of tacrolimus in combination with mycophenolic acid and prednisone. The human leukocyte antigen (HLA) A, B, and DR for both recipient and donor and the number of HLA mismatches were registered. Other variables recorded were amylase, lipase, blood glucose, hemoglobin A1C (HbA1C), c-peptide, glutamic acid decarboxylase antibodies (antiGAD), blood type, surgical technique (duo-duodenal or duo-jejunal anastomosis), diabetes mellitus type and vintage, renal replacement therapy at time of transplantation (peritoneal dialysis, hemodialysis, non-dialysis dependent chronic kidney disease), dialysis vintage, graft perfusion solution, and post-transplant surgical reintervention.

### Time and Tests Below the Therapeutic Range

The first tacrolimus dose was administered pre-transplantation as part of the induction immunosuppression protocol, and the first levels were drawn 48 h after surgery. The minimum tacrolimus levels targeted were 10 ng/mL during the first 3 months, 8 ng/mL between the third and sixth month, and 6 ng/mL afterward. The percentage of time below the tacrolimus therapeutic range was calculated by adding the number of days below the target and dividing them by the total number of monitored days. Likewise, the percentage of the number of tests below the therapeutic range was calculated by adding the number of test results below the target and dividing them by the total number of tests performed respectively [24].

### Biopsy-Proven Rejection and DSA Determination

Pancreatic graft biopsies were conducted per protocol (3 weeks and 12 months after transplantation) or per cause. According to the center’s guidelines, biopsies prompted per cause were indicated when patients exhibited a consistent rise (on at least two occasions, with a gap of more than 48 h) in pancreatic enzymes (amylase and/or lipase) exceeding three times the upper normal levels, developed dnDSA, or persistent hyperglycemia (fasting blood glucose >120 mg/dL on more than two determinations). Tissue samples were collected using a percutaneous needle puncture guided by ultrasound, and their histological categorization followed the criteria outlined in the 2011 Banff classification [32]. Tacrolimus trough levels, amylase, lipase, c-peptide, HbA1C, and anti-HLA and antiGAD antibodies were determined at the time of biopsy. *De novo* DSAs were defined as HLA antibodies against the donor that were absent before transplantation. DSAs were characterized by having a mean fluorescence intensity (MFI) that was more than double the negative control’s value and an absolute MFI exceeding 500 [33]. The MFIs were adjusted based on the manufacturer’s guidelines by comparing them to the negative control beads.

### Statistical Methods

For data following normal distribution, quantitative variables are presented as mean and standard deviation; otherwise, they are presented as median and interquartile range. Categorical variables are described in terms of absolute and relative frequencies. The normality of quantitative variables was assessed using the Shapiro-Wilk test and Q-Q plots. When the data was not normally distributed, a U-Mann Whitney test was employed for the quantitative variables’ comparison between the two groups; for normally distributed data, an independent Student’s t-test was used instead. Disparities in categorical variables were evaluated using the χ2 test, while Fisher’s exact test was utilized when a category contained fewer than five occurrences. Receiver Operating Characteristic (ROC) curves were generated, and metrics such as sensitivity, specificity, positive predictive value (PPV), and negative predictive value (NPV) were calculated. A *p*-value of less than 0.05 was considered statistically significant. All analyses were conducted using IBM SPSS^®^ Statistics version 26.

## Results

### Participants

One hundred and thirty-one patients were included; sixty-nine (52.7%) were male, 122 (93.1%) were SPK, and nine were PAK. One hundred and twenty-nine (98.5%) had type 1 diabetes, one had type 2 diabetes, and one had diabetes after a necrotizing hemorrhagic pancreatitis. Seventy (53.4%) of the donors were male. Sixty-four patients (48.9%) received basiliximab, and 65 (49.6%) received rATG as induction immunosuppression. All patients were on mycophenolate and tacrolimus as maintenance treatment. The median follow-up was 104 (45.5–139) months. Recipient and donor characteristics are detailed in Table 1.

**TABLE 1:** Differences in clinical and analytical variables between groups.

Variable	Biopsy-proven rejection	No rejection	*p*-value
	N = 16	N = 115	
Pancreas transplantation type, n (%)			0.045
SPK	13 (10.66)	109 (89.34)	
PAK	3 (33.33)	6 (66.67)	
Indication for pancreas transplantation, n (%)			0.868
Type 1 diabetes	16 (12.4)	113 (87.6)	
Type 2 diabetes	0	1	
Hemorrhagic pancreatitis	0	1	
Male donor, n (%)	9 (12.85)	61 (87.14)	0.810
Lipase, U/L, median (IQR)	96 (161)	41 (39.75)	0.078
Amylase U/L, median (IQR)	111.5 (99.5)	88.5 (49.75)	0.111
Glucose mg/dL, median (IQR)	92.5 (38.25)	87.5 (14)	0.284
HbA1C (%), median (IQR)	5.35 (0.93)	5.5 (0.65)	0.894
antiGAD U/mL, median (IQR)	0.55 (15.73)	0.6 (3.72)	0.692
C-peptide ng/mL, mean ± SD	3.4 ± 1.81	3.52 ± 2.53	0.807
Recipient age, mean ± SD	39.66 ± 9.41	41.35 ± 7.53	0.281
Donor age, median (IQR)	36 (23)	36 (14.5)	0.430
BMI, kg/m^2^	23.9 ± 2.6	23.35 ± 3.03	0.488
Diabetes vintage (months), mean ± SD	25.7 ± 9.59	27.19 ± 8.21	0.982
Intrapatient variability, median (IQR)	52.18 (0.32)	43.3 (0.13)	0.809
Time BTR, median (IQR)	37.98 (0.37)	20.45 (0.26)	0.028
Tests BTR, mean ± SD	42.43 ± 13.81	32.5 ± 17.07	0.013

antiGAD, Anti-glutamic acid decarboxylase antibody; BMI, body mass index; BTR, below therapeutic range; HbA1C, hemoglobin A1C; PAK, pancreas after kidney; SPK, simultaneous pancreas kidney.

### Clinical Outcomes

The median time and tests below the therapeutic range for the entire group were 24.44% (8.58%–40.52%) and 28.6% (20.26%–42.45%), respectively, with a median IPV of 45.15% (38.87%–55.56%). Overall, 32.8% and 35.1% of patients had tacrolimus levels below the therapeutic range in more than 36% of the tests performed and more than 31.5% of the time. Eighteen (13.74%) patients died, nine (50%) of them with a functioning graft. Thirty patients lost their pancreatic graft function (22.9%), with a median survival time of 101.8 (67.6–119.9) months.

Out of the 16 instances of pancreas BPAR (12.2%), two occurred in the same patient. Two were antibody-mediated rejections, while the remaining 14 were T-cell-mediated, with 11 being grade I, 2 grade II, and 1 grade III. The mean BPAR-free survival time was 85.24 ± 17.38 months. Lipase levels were higher in patients with BPAR (185.25 ± 264.37 vs. 59.77 ± 47.92 U/L in those without rejection, *p* = 0.001). There was a trend towards a higher incidence of rejection between PAKs and SPKs (3, 50% vs. 13, 11.9%; OR 4.19, 0.935–18.798; *p* = 0.045).

Patients with BPAR had a significantly higher time (39.9% ± 24% vs. 25.72 %± 21.57%, *p* = 0.016) and number of tests (41.95% ± 13.57% vs. 29.96% ± 17.33%, *p* = 0.009) below therapeutic range compared to those without rejection. There was no association between tacrolimus’ IPV or amylase with pancreas BPAR incidence (48.7% vs. 49.9%, *p* = 0.81; 125.63 vs. 98.9, *p* = 0.11).

**FIGURE 2:**
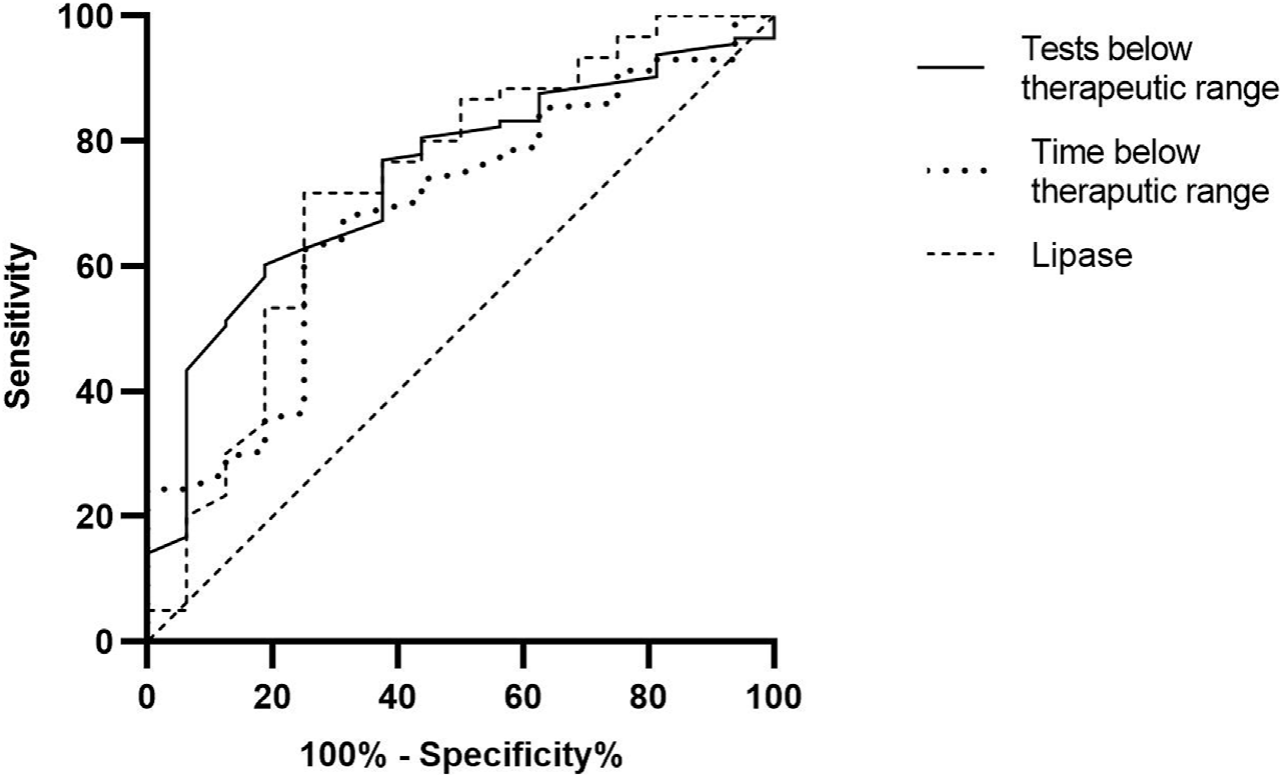
Receiver operating characteristic (ROC) curves of evaluated tests.

The area under the curve (AUC) for time and tests below the therapeutic range and BPAR incidence were 70.5% and 73.2%, respectively, and 72.9% for lipase and 63.2% for amylase (Figure 2). Based on the highest sensitivity and specificity coordinates, we evaluated the former in two categories: 31.5% for time and 34% for tests. Patients who maintained tacrolimus levels more than 31.5% of the time below the therapeutic range until the moment of the biopsy had a significantly higher probability of having a BPAR (22.1% vs. 6%, *p* = 0.004; OR 4.629, 1.502–14.286) than those who did not. This test had a sensitivity of 68.75%, a specificity of 67.83%, a PPV of 22.92%, and an NPV of 93.98%. Similarly, patients with 36% or more tacrolimus tests below the therapeutic range had a higher probability of pancreas BPAR (23.3% vs. 6.9%, *p* = 0.008; OR 4.098, 1.375–12.195). This test had a sensitivity of 62.5%, a specificity of 71.05%, a PPV of 23.26%, and an NPV of 93.1%. On the other hand, lipase had a specificity of 98.33% and a similar NPV of 89.70%. In this case, also based on the ROC coordinates, we divided the set with a lipase cutoff of 53 U/L. A lipase higher than this correlated with an increased risk of pancreas BPAR (41.4% vs. 8.5%, *p* = 0.001; OR 7.588, 2.145–26.839) with a sensibility of 68.75%, a specificity of 67.83%, a PPV of 22.92%, and an NPV of 93.98%.

Eleven (8.4%) patients developed dnDSAs. Among them, eight recipients had dnDSAs from class II, two from class I, and one from both class I and II. Of these antibodies, 6 (46.1%) were HLA-DQ, 4 (30.78%) were HLA-DR, and 3 (23.08%) were HLA-A. There was a non-significant difference with tacrolimus’ IPV (47.9% ± 14.44% vs. 69.27% ± 44.76%, *p* = 0.193), amylase (116 ± 42.54 vs. 108 ± 50.23 U/L, *p* = 0.68), and lipase (234.67 ± 344.53 vs. 83.83 ± 71.46 U/L, *p* = 0.23).

The time and tests below the therapeutic range were associated with an increased incidence of dnDSA development (30.18% vs. 43.93%, *p* = 0.017 and 40.57% vs. 71.26%, *p* = 0.048, respectively). However, the AUC for the time was smaller than for the number of tests below the therapeutic range (66.2% vs. 71.3%). When analyzing both variables as dichotomic based on the cutoff values established previously, only the number of tests remained statistically significant (38.9% vs. 9.4%, *p* = 0.012; OR 6.135, 1.346–27.78).

## Discussion

In this study, we determined that patients who spent longer time or had more tests below the tacrolimus therapeutic range had an increased incidence of acute pancreatic graft rejection. We also found that a cutoff of 31.5% of the time and 34% of the tests were significantly associated with an increased pancreatic rejection incidence with a very high specificity and NPV. Moreover, those with a higher number of tests below the therapeutic range were also associated with an increased incidence of dnDSA. Finally, we performed ROC analysis and found that the time and tests below the therapeutic range had a similar area under the curve compared to lipase for pancreatic graft BPAR.

Tacrolimus is a crucial part of maintenance immunosuppression in pancreas transplantation and is recommended by current guidelines despite being prescribed off-label due to lack of approval by the US Food and Drug Administration. However, sufficient evidence has proven its efficacy in improving short- and long-term pancreatic graft survival [34]. There is some data on specific dosing of immunosuppressors and dnDSA formation in pancreas transplantation. Yet, data on the impact of dosage and monitoring trough levels on the risk of rejection is lacking. That becomes of great importance as tacrolimus has a limited therapeutic threshold that needs to be constantly adjusted by transplant professionals. In this sense, there is evidence that associates the time spent within the therapeutic range of tacrolimus and various solid-organ graft survival, such as lung, heart, and kidney [35]. Nevertheless, there is currently no data on this subject in patients with SPK or PAK.

In our cohort, we found that around a third of patients were below the targeted tacrolimus therapeutic range, similar to data published by Davis et al. [33], although theirs is only from the first-year post-kidney transplantation.

Regarding BPAR, we found a rejection incidence of 12.2%, similar to the reported 10%–14% incidence published previously [36]. There is evidence evaluating the usefulness of TTR in other solid organ transplants, such as lung transplantation, where a cutoff of 30% or an increase of 10% of the baseline TTR, in turn, decreased the risk of graft rejection [20, 28]. Similarly, a study on living kidney donors determined that a TTR below 22% increased the risk of kidney graft rejection [26], while another one with deceased donors determined a cutoff of 30% [25]. In the case of heart transplants, a TTR lower than 25% was associated with more rejections [29]. In our case, we found that spending more than 31.5% of the TTR and more than 34% of the tests below the therapeutic level was significantly associated with an increased risk of acute rejection. We also found that an elevated lipase above 53 UI/L was significantly associated with an increased incidence of BPAR. To our knowledge, this is the first evidence on a specific lipase cutoff value since there is no evidence on this subject for stable pancreatic transplant recipients beyond the early postoperative scenario, where they associate a mean value of 634 ± 247 UI/L with an increased incidence of BPAR [37].

Regarding humoral response, there is evidence that lower tacrolimus levels are associated with a higher risk of dnDSA development in kidney transplant recipients [25]. The mentioned article by Davis et al. [33] found dnDSAs in 21.7% of their cohort. Their appearance was associated with a time outside the therapeutic range of tacrolimus greater than 30%. In our cohort, 8.4% of patients developed dnDSA, and a percentage of 36% or more of tests below the therapeutic range increased the risk of developing them [37]. We did not find any significant association with lipase blood levels. This may be explained by the fact that lipase only translates to an ongoing graft injury, and while a patient with dnDSAs is at risk for rejection, it may not have occurred yet, hence there is no injury. Additionally, not all rejections are antibody-mediated, which may also explain the presence of a significant BPAR and not a dnDSA association.

A study by Torabi et al. [38] performed on pancreatic transplant recipients showed that extended-release tacrolimus was associated with fewer rejections and non-significantly with less IPV. The study by Davis et al. [25] in kidney and SPK recipients determined that an IPV greater than 44% was associated with increased dnDSA development. However, they did not perform an SPK subanalysis. In contrast, we did not find an association between IPV and BPAR and only found a non-significant difference with increased IPV and dnDSA formation.

This study has several limitations. There is a possibility that certain tacrolimus levels may not accurately represent trough levels. Concurrent medical conditions or medications may influence levels, and we did not perform a subanalysis according to patients’ baseline immunological risk, given the small sample we worked with. Also, as were only evaluating immunological outcomes, we decided only to study the time below and not within the therapeutic range. Finally, this is a single-center retrospective study, which limits our capacity to determine the exact number of per cause and protocol biopsies, the interpretation of the results obtained, and their generalizability.

To conclude, this study highlights the significance of maintaining proper levels of immunosuppression in pancreas transplantation. It suggests that identifying patients at risk of rejection can potentially be done by monitoring the percentage of tests that fall below the therapeutic range. Additionally, this method could prove to be a valuable tool if combined with new rejection markers, such as donor-derived cell-free DNA [39]. This would enable identification of high-risk patients for immunological exposure, while also allowing for detection of graft damage, without incurring additional financial expenses.

## Data Availability

The raw data supporting the conclusion of this article will be made available by the authors, without undue reservation.

## Abbreviations

antiGAD, glutamic acid decarboxylase antibodies; AUC, area under the curve; BPAR, biopsy-proven acute rejection; dnDSA, *de novo* donor-specific antibody; HbA1c, hemoglobin A1C; HLA, human leukocyte antigen; INR, international normalized ratio; IPV, intrapatient variability; MFI, mean fluorescence intensity; NPV, negative predictive value; PAK, pancreas after kidney; PPV, positive predictive value; rATG, rabbit antithymocyte globulin; ROC, receiver operating characteristic; SPK, simultaneous pancreas-kidney; TTR, time in therapeutic range.
